# Supplementary material for: Validation of a Rapid Multiresidue Method for the Determination of Pesticide Residues in Vine Leaves. Comparison of the Results According to the Different Conservation Methods
Source: Molecules. 2021 Feb 22;26(4):1176. doi: 10.3390/molecules26041176 (PMC7927040; doi:10.3390/molecules26041176)
Supplement: Supplementary file 1 [file molecules-26-01176-s001.pdf]

Table S1. List of the 33 pesticide residues analyzed in grapevine leaves, use type, chemical group and mode of action.

Molecule	CAS Number	Formula	Log P (Kow)	Chemical group	Use Type	Mode of action
Acetamiprid	135410-20-7	C10H11ClN4	0.80	Neonicotinoid	Insecticide	Systemic
Azoxystrobin	131860-33-8	C22H17N3O5	2.50	Strobilurin	Fungicide	Systemic
Bifenthrin	82657-04-3	C23H22ClF3O2	6.00	Pyrethroid	Acaricide, Insecticide	Contact
Boscalid	188425-85-6	C18H12Cl2N2O	2.96	Carboxamide	Fungicide	Systemic
Carbendazim	10605-21-7	C9H9N3O2	1.52	Benzimidazole	Fungicide	Systemic
Chlorpyriphos	2921-88-2	C9H11Cl3NO3PS	4.96	Organophosphorous	Insecticide	Contact
Cypermethrin	52315-07-8	C22H19Cl2NO3	5.55	Pyrethroid	Insecticide	Contact
Cyproconazole	113096-99-4	C15H18ClN3O	2.90	Triazole	Fungicide	Systemic
Cyprodinil	121552-61-2	C14H15N3	3.59	Anilinopyrimidine	Fungicide	Systemic
Deltamethrin	52918-63-5	C22H19Br2NO3	4.60	Pyrethroid	Insecticide	Contact
Diazinon	333-41-5	C12H21N2O3PS	3.81	Organophosphorous	Acaricide, Insecticide	Contact
Difenoconazole	119446-68-3	C19H17Cl2N3O3	4.40	Triazole	Fungicide	Systemic
Dimethoate	60-51-5	C5H12NO3PS2	0.78	Organophosphorous	Acaricide, Insecticide	Systemic
Fenazaquin	120928-09-8	C20H22N2O	5.51	Quinazoline	Acaricide	Contact
Fenhexamid	126833-17-8	C14H17Cl2NO2	3.51	Hydroxyanilide	Acaricide	Translaminar
Hexaconazole	79983-71-4	C14H17Cl2N3O	3.90	Triazole	Fungicide	Systemic
Imazalil	35554-44-0	C14H14Cl2N2O	3.82	Imidazole	Fungicide	Systemic
Imidacloprid	138261-41-3	C9H10ClN5O2	0.57	Neonicotinoid	Insecticide	Systemic
Indoxacarb	173584-44-6	C22H17ClF3N3O7	4.65	Oxadiazine	Insecticide	Contact
Kresoxim-methyl	143390-89-0	C18H19NO4	3.40	Strobilurin	Fungicide	Translaminar
Lambda- Cyhalothrin	91465-08-6	C23H19ClF3NO3	7.00	Pyrethroid	Insecticide	Contact
Lufenuron	103055-07-8	C17H8Cl2F8N2O3	5.12	Benzoylurea	Acaricide, Insecticide	Systemic
Metalaxyl	57837-19-1	C15H21NO4	1.65	Phenylamide	Fungicide	Systemic
Myclobutanil	88671-89-0	C15H17ClN4	2.94	Triazole	Fungicide	Systemic
Penconazole	66246-88-6	C13H15Cl2N3	3.72	Triazole	Fungicide	Systemic
Propargite	2312-35-8	C19H26O4S	5.70	Sulphite ester	Acaricide	Contact
Propiconazole	60207-90-1	C15H17Cl2N3O2	3.72	Triazole	Fungicide	Systemic
Pyraclostrobin	175013-18-0	C19H18ClN3O4	3.99	Strobilurin	Fungicide	Translaminar
Pyridaben	96489-71-3	C19H25ClN2OS	6.37	Pyridazone	Acaricide, Insecticide	Contact
Pyrimethanil	53112-28-0	C12H13N3	2.84	Anilinopyrimidine	Fungicide	Translaminar
Tebuconazole	107534-96-3	C16H22ClN3O	3.70	Triazole	Fungicide	Systemic
Tetraconazole	112281-77-3	C13H11Cl2F4N3O	3.56	Triazole	Fungicide	Systemic
Trifloxystrobin	141517-21-7	C20H19F3N2O4	4.50	Strobilurin	Fungicide	Translaminar

Table S2. Precursor, Transition ions and Source Parameters for the 33 pesticide residues analyzed by the LC-MS/MS method.

Condition	Content
Instrument	Model AB Sciex 3200 QTRAP LC-MS/MS SYSTEM
Column	C_{18} column, Phenomenex Analytical Synergi, 150 x 2 mm, 2.5 μm particle size
Column Flow	Gradient elution program at 0.4 ml/min
Source temperature Ion Spray- Potential	500°C - 5000v
Mode	Electron Spray Ionization, Positive Mode

Trade name	RT	Transition	DP	CE	CXP	Transition	DP	CE	CXP	LOD	LOQ
	(min.)	Q1 (m/z)	(Volts)			Q2 (m/z)	(Volts)			(ng/g)	
Acetamiprid	10.07	223.0 --> 126.0	36	26	7	223.0 --> 99.1	36	47	4	1.85	6.18
Azoxystrobin	13.57	404.1 --> 372.1	36	19	20	404.1 --> 344.1	36	29	18	1.70	5.68
Bifenthrin	22.87	440.1 --> 181.2	41	19	14	440.1 --> 166.0	41	59	14	1.43	4.72
Boscalid	14.27	343.1 --> 307.1	86	29	8	343.1 --> 140.0	86	31	10	2.05	6.85
Carbendazim	7.67	192.0 --> 160.0	40	25	6	192.0 --> 132.0	36	23	4	1.38	4.55
Chlorpyriphos	21.27	350.0 --> 198.0	21	25	3	350.0 --> 96.9	21	25	3	1.21	4.04
Cypermethrin	21.97	416.1 --> 191.9	36	27	18	416.1 --> 127.0	36	43	0	1.83	6.11
Cyproconazole	14.97	292.1 --> 70.2	25	18	4	292.1 --> 125.1	66	30	10	0.84	2.77
Cyprodinil	16.67	226.0 --> 93.0	61	45	4	226.0 --> 77.0	61	45	4	2.36	7.88
Deltamethrin	22.87	506.9 --> 181.3	51	23	8	506.9 --> 280.6	51	51	14	1.23	4.06
Diazinon	17.97	305.1 --> 169.1	25	22	4	305.1 --> 153.0	36	31	4	0.89	2.94
Difenoconazole	19.27	406.2 --> 251.1	96	37	14	406.2 --> 337.0	96	37	14	1.83	6.11
Dimethoate	10.67	230.0 --> 125.0	76	10	10	230.0 --> 199.1	81	12	6	1.97	6.58
Fenazaquin	23.27	307.0 --> 161.0	41	25	10	307.0 --> 147.0	66	29	12	2.03	6.70
Fenhexamid	15.67	302.0 --> 97.1	51	25	12	302.0 --> 55.0	81	19	4	1.23	4.06
Hexaconazole	18.27	314.0 --> 70.0	40	10	15	314.0 --> 159.0	40	10	15	1.69	5.64
Imazalil	10.97	297.0 --> 201.0	26	31	6	297.0 --> 159.0	46	27	4	2.43	8.02
Imidacloprid	9.47	256.0 --> 209	51	21	7	256.0 --> 175.0	46	25	7	1.93	6.45
Indoxacarb	20.47	528.0 --> 203.0	76	51	7	528.0 --> 56.0	76	51	7	1.33	4.44
Kresoxim-methyl	17.57	314.0 --> 116.1	66	19	8	314.0 --> 206.0	66	11	18	1.91	6.38
Lambda-Cyhalothrin	22.37	467.1 --> 225.0	41	23	8	467.1 --> 141.1	35	30	10	1.26	4.16
Lufenuron	20.67	511.1 --> 158.1	96	31	12	511.1 --> 141.0	96	67	10	1.83	6.11
Metalaxyl	11.87	280.2 --> 220.2	71	10	10	280.2 --> 192.3	76	10	10	1.86	6.21
Myclobutanil	15.07	289.0 --> 70.0	30	18	28	289.0 --> 125.0	56	30	4	1.19	3.93
Penconazole	17.47	284.0 --> 159.0	40	10	15	284.0 --> 125.0	16	10	6	2.14	7.15
Propargite	21.87	368.0 --> 231.0	20	11	14	368.0 --> 175.3	36	23	4	1.23	4.06
Propiconazole	17.87	342.1 --> 159.0	35	30	12	342.1 --> 69.1	86	43	4	1.43	4.72
Pyraclostrobin	18.47	388.0 --> 163.0	25	25	6	388.0 --> 194.0	26	29	4	1.53	5.05
Pyridaben	23.17	365.0 --> 309.0	56	19	8	365.0 --> 147.0	51	37	10	1.31	4.32
Pyrimethanil	12.77	200.0 --> 107.0	45	31	4	200.0 --> 82.1	46	35	4	1.64	5.41
Tebuconazole	17.67	308.0 --> 70.0	30	24	4	308.0 --> 125.0	41	47	10	1.62	5.35
Tetraconazole	17.17	372.0 --> 158.9	35	35	12	372.0 --> 70.0	81	45	4	2.23	7.36
Trifloxystrobin	19.57	409.0 --> 186.0	25	23	4	409.0 --> 206.0	26	19	4	1.18	3.89

RT: retention time, **CE**: collision energy, **DP**: declustering potential, **EP**: entrance potential, **CXP**: collision cell exit potential, **Q1**: quantifier transition, **Q2**: qualifier transition 2.
